# Spatial and molecular resolution of diffuse malignant mesothelioma heterogeneity by integrating label-free FTIR imaging, laser capture microdissection and proteomics

**DOI:** 10.1038/srep44829

**Published:** 2017-03-30

**Authors:** Frederik Großerueschkamp, Thilo Bracht, Hanna C. Diehl, Claus Kuepper, Maike Ahrens, Angela Kallenbach-Thieltges, Axel Mosig, Martin Eisenacher, Katrin Marcus, Thomas Behrens, Thomas Brüning, Dirk Theegarten, Barbara Sitek, Klaus Gerwert

**Affiliations:** 1 Ruhr-University Bochum, Department of Biophysics, Bochum, Germany; 2 Ruhr-University Bochum, Medizinisches Proteom- Center (MPC), Bochum, Germany; 3 Institute for Prevention and Occupational Medicine of the German Social Accident Insurance, Institute of the Ruhr Universität Bochum (IPA), Bochum, Germany; 4 University Duisburg Essen, University Hospital Essen, Institute of Pathology, Essen, Germany

## Abstract

Diffuse malignant mesothelioma (DMM) is a heterogeneous malignant neoplasia manifesting with three subtypes: epithelioid, sarcomatoid and biphasic. DMM exhibit a high degree of spatial heterogeneity that complicates a thorough understanding of the underlying different molecular processes in each subtype. We present a novel approach to spatially resolve the heterogeneity of a tumour in a label-free manner by integrating FTIR imaging and laser capture microdissection (LCM). Subsequent proteome analysis of the dissected homogenous samples provides in addition molecular resolution. FTIR imaging resolves tumour subtypes within tissue thin-sections in an automated and label-free manner with accuracy of about 85% for DMM subtypes. Even in highly heterogeneous tissue structures, our label-free approach can identify small regions of interest, which can be dissected as homogeneous samples using LCM. Subsequent proteome analysis provides a location specific molecular characterization. Applied to DMM subtypes, we identify 142 differentially expressed proteins, including five protein biomarkers commonly used in DMM immunohistochemistry panels. Thus, FTIR imaging resolves not only morphological alteration within tissue but it resolves even alterations at the level of single proteins in tumour subtypes. Our fully automated workflow FTIR-guided LCM opens new avenues collecting homogeneous samples for precise and predictive biomarkers from omics studies.

Protein biomarkers are promising biological indicators of cancer status, and they allow clinicians to monitor the safety and efficacy of novel therapeutic agents^1^,^2^,^3^. However, the identification of specific and sensitive biomarkers for a given cancer is still a challenge; especially for biomarkers used in early detection and assessment of prognosis^4^,^5^. Although numerous proteome studies seeking to identify new cancer biomarkers have been performed in recent decades, their success in improving cancer diagnosis remains marginal^6^.

Many of these studies failed due to the challenge of tissue heterogeneity. The isolation and analysis of the affected cell types is essential for the detection of reliable biomarkers with high specificity^7^,^8^. To understand tumour heterogeneity, first spatially resolved annotation of the tissue is necessary and then molecular resolving analysis of the annotated region of interest (ROI)^9^. How this challenge can be addressed is shown here exemplified by diffuse malignant mesothelioma (DMM) of the pleura parietalis and visceralis. It is a rare and histologically heterogeneous cancer with three subtypes: epithelioid, sarcomatoid and biphasic. DMM is caused primarily by exposure to asbestos^10^. The gold standard for DMM diagnosis is histological confirmation, including immunohistochemical panels^11^. Its frequent phenotypic heterogeneity and diverse architectural patterns complicate spatially resolved molecular analysis of DMMs. Furthermore, mesothelioma sometimes mimic other neoplasms, notably adenocarcinoma of the lung and sarcomas^12^. Furthermore, beside the histological heterogeneity the small number of patients makes it difficult to study DMM in detail^13^.

In order to regard the heterogeneity of tissue samples and to study the biomarker content of individual subpopulations within the tumour microenvironment, laser capture microdissection (LCM) is successfully applied in proteomics-based biomarker discovery^14^,^15^. Initially, histological staining or immunohistochemistry (IHC) was conducted on a thin-section, and identified ROIs were transferred to an unstained adjacent section of the tissue. In general, there is an uncontrollable mismatch between the ROI identified through an IHC marker and the dissected ROI in the adjacent section. Subsequent research showed that tissue samples can be stained without compromising the yield and quality of RNA or proteins^16^,^17^,^18^. Both approaches for ROI identification require a highly specialized skill set for morphological recognition of the tissue of interest, which is inherently subject to effects inter- and intra-observer variation^19^. This becomes an evident problem in multicentre studies where several experts have to decide which ROIs were chosen. Attempts to minimize this variation have been reported by extensive standardized training of the analysts, yet failing to fully eliminate observer variance^20^,^21^. Furthermore, several stains and IHC marker have to be used to visualize every aspect of a heterogeneous tissue like lung and pleural neoplasms. The decision is biased by the amount of embedded tissues, expert knowledge, and the used stains or IHC markers.

The novel approach, FTIR-guided LCM for proteomics, presented here achieves high-quality and unbiased results with no inter- and intra-observer variability in sample selection by replacing the histologic staining and IHC with label-free Fourier transform infrared (FTIR) imaging tissue annotation. In recent years, FTIR imaging has shown its potential in tissue classification. The robust and objective results produced by FTIR imaging minimized inter- and intra-observer variability and allow for automated, spatially resolved label-free annotation of tissue samples^22^,^23^,^24^,^25^,^26^,^27^. By FTIR imaging, vibrational spectra of unstained tissue thin-sections are measured at high spatial resolution. Each vibrational spectrum functions as a molecular and structural fingerprint for the underlying tissue, which can be used for automated classification of tissue types^28^,^29^,^30^. Even subtle changes occurring on molecular or structural level in tissue caused by a disease can be resolved^31^,^32^,^33^. The analysis is performed on the untreated sample without the addition of chemical stains or reagents.

We have presented success with FTIR imaging tissue annotation in previous studies on the automated classification of colorectal cancer (accuracy of 96%, sensitivity of 94%, and specificity of 100%)^34^ and the differential diagnosis of the cancer, as well as its grading resulting in high accuracy, sensitivity, and specificity (G1: 99%, 94%, 100%; G2: 86%, 83%, 94%; G3: 87%, 89%, 87%)^35^. Furthermore, we analysed lung tumours and thoracal tumours, showing that differential diagnosis down to the subtype of adenocarcinomas of the lung is possible using FTIR imaging (accuracy of 96%; no binary classification so sensitivity and specificity cannot be calculated)^36^.

Here, we extended our FTIR imaging approach for lung and thoracal tumours to resolve the tissue heterogeneity of DMM tissue samples from the pleura parietalis and visceralis for subsequent proteome analysis. All tissue types are differentiated simultaneously and label-free starting with the pathologic regions, following the tumour type (e.g. metastases of lung tumours in the pleura, or DMM)^36^, and finally the tumour subtype (e.g. epithelioid, biphasic, or sarcomatoid for DMMs). The spatially resolved, automated, label-free FTIR imaging results were then transferred to LCM for harvesting of label-free, homogeneous tissue samples from heterogeneous tissue. The collected samples were then used for proteome analysis to determine protein biomarkers for the heterogeneity between the two subtypes (epithelioid and sarcomatoid). The differentially expressed proteins were compared to already known or previously described markers used in the clinical IHC panel. The novel FTIR-guided LCM approach presented here allows for the first time harvesting of label-free, homogeneous, native tissue samples from heterogeneous cancer tissue for subsequent proteome studies considering simultaneously all tissue types in an automated approach without further annotation through an expert.

## Results

### Workflow design of FTIR-guided LCM

**Figure 1:**
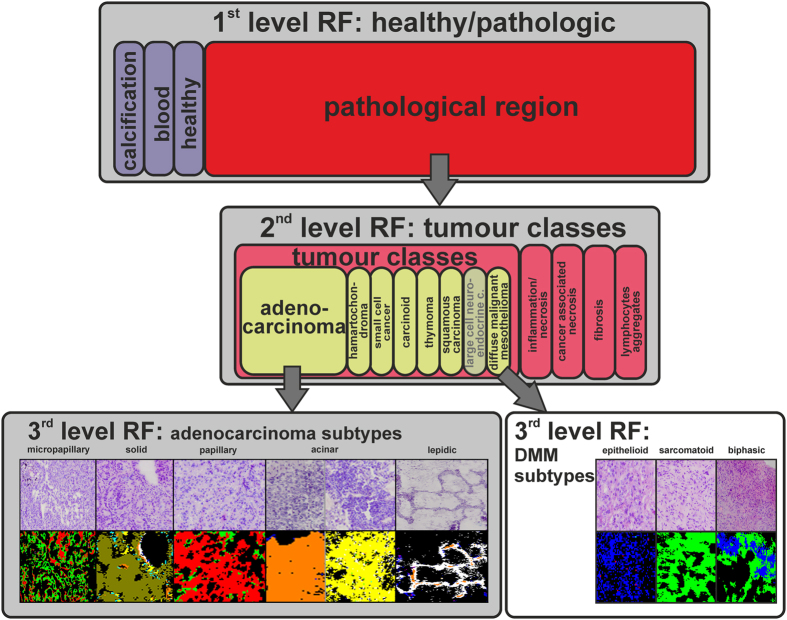
Schematic workflow of the differential FTIR imaging tissue characterization for thoracal tumours. Tissue annotation by FTIR imaging for lung tumours and thoracal tumours is illustrated. The classifiers for lung tumours and thoracal tumours (shaded in grey) were previously published and reprinted from Großerueschkamp *et al*. Analyst 2015^36^. In a first classifier, the pathologic regions were identified. In a second classifier, the different tumour classes of the pathologic region are differentiated. Finally in the third classifier, a differential diagnosis of the tumour subtypes is provided. The DMM subtype classifier was trained especially for this study based on newly acquired samples. This is an important step establishing a differential and predictive thoracal tissue classifier.

**Figure 2:**
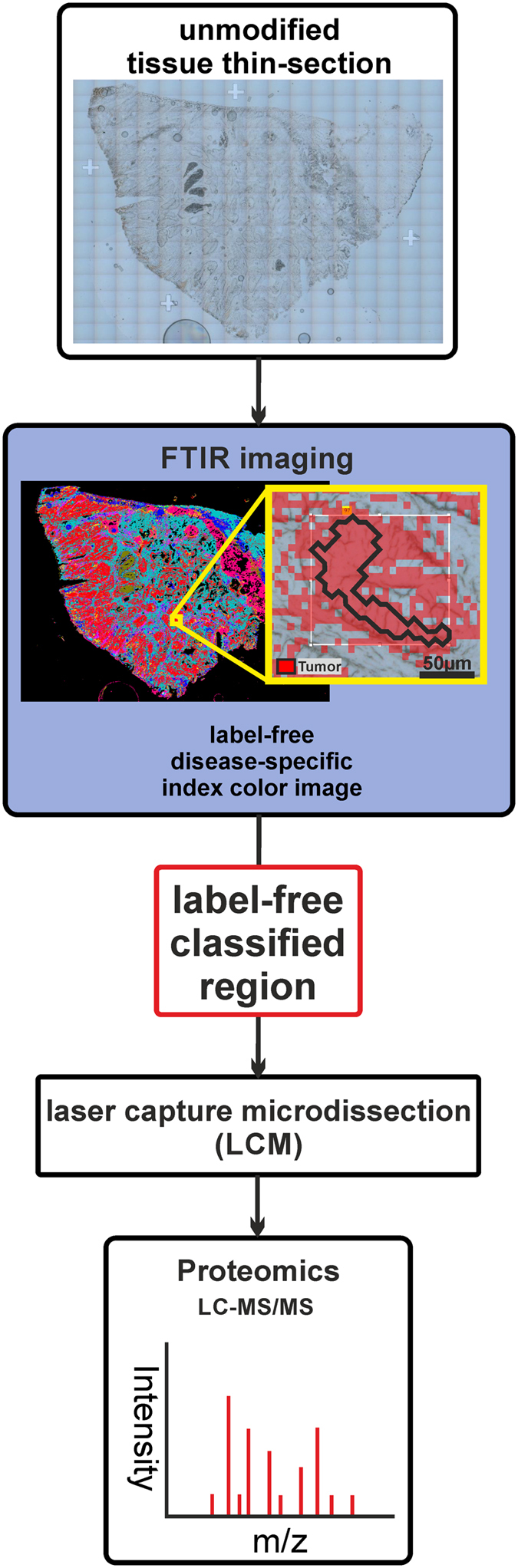
Schematic workflow of the novel label-free approach (FTIR-guided LMD). Today sample collection via LCM can only be done on labelled or adjacent unlabelled tissue thin-sections. The results gathered with this current method can be influenced by modification or transfer errors in adjacent section for later -omics studies. In contrast FTIR-guided based tissue classification can characterize label-free tissue thin-sections with high spatial accuracy. This novel characterization method can be used for collecting samples for subsequent -omics analyses from the same, well classified unlabelled thin-section. While the method was demonstrated here with subsequent proteome analysis, other -omics techniques can be used as well.

**Figure 3:**
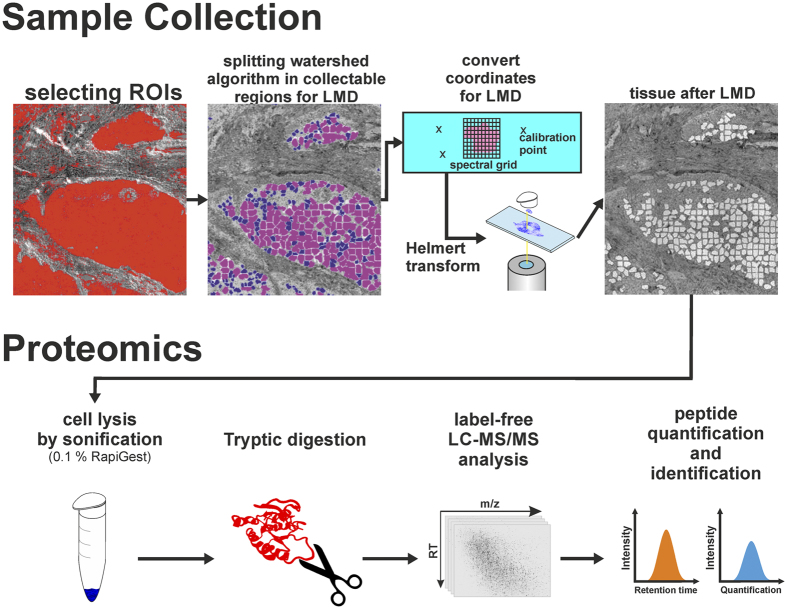
Coordinate transfer between FTIR imaging and laser capture microdissection steps for subsequent proteome analysis. Shown is a schematic overview of sample collection using FTIR imaging. In the sample collection stage, the ROIs were split into smaller regions for LCM dependent on the used magnification. The coordinates of the cutting shapes were transferred to the LCM microscope using a two-dimensional Helmert transformation with three calibration points. The samples were collected for proteome analysis. The samples were sonicated to lyse the cells, digested with Trypsin to obtain peptides, and analysed by LC-MS/MS. Data analysis provides the final results.

For label-free collection of specific tissue samples from heterogeneous tissue for proteome analysis, we integrate label-free FTIR imaging of native tissue thin-sections with LCM. This new approach allowed us to identify ROIs from tissue thin-sections automatically without relying on any labels or subsequent visual inspection to collect them precisely using LCM. We placed 10-μm-thick cryosections on polyethylene terephthalate (PET) frame slides without further processing. Afterwards, the sections were measured by FTIR imaging from 2700–950 cm^−1^ under dry air conditions. The resulting spectral data were pre-processed and annotated by a set of previously trained random forest classifiers (Fig. 1)^36^,^37^. In the presented study the previously published set of classifiers (shaded in grey in Fig. 1, partly reprinted from Großerueschkamp *et al*. Analyst 2015^36^) was extended by a novel classifier for DMM subtypes which was trained on newly acquired tissue samples. From these automated, spatially resolved, and label-free annotated tissue samples, ROIs were selected and transferred to LCM for automated dissection (Fig. 2). The ROIs were selected based on the annotation by the random forest classifier (for details see the ‘coordinate transfer’ section under ‘material and methods’)^34^,^36^. The coordinates of the selected ROIs were transformed to the LCM by a 2-dimensional Helmert transformation. The spatial accuracy is around 10 μm with the used setup. Then, the ROIs were automatically dissected and collected for label-free proteome analysis (Fig. 3). Usually, total areas of the same size were collected for each tissue sample by LCM for proteome analysis to obtain equal protein distribution. The presented novel approach estimates the relative protein concentration based on the integral of the protein backbone vibration (amide I band). Each measured amide I band in the spectra is proportional to a concentration according to the Lambert-Beer law. Thus, samples of the same protein concentration will show the same overall integral amid I band (~1654 cm^-1^). The use of FTIR-guided LCM allows the user to monitor the equal distribution of relative protein concentrations in the samples based on IR absorbance without additional working steps.

The pixel resolution of FTIR imaging can reach about 1.7 μm with high-magnification, NA 0.81, 25 × objective on an Agilent Cary 620 with oversampling. But because fresh tissue can degrade rapidly, we decided to optimize measurement time and rather sacrifice spatial resolution. Therefore, a 15 × objective (NA 0.62) was used with a spatial resolution of ~10 μm. The high spatial accuracy of this method is demonstrated with an USAF (United States Air Force) resolution target (Fig. S1A, USAF 1951 1X, Edmund Optics, NJ, USA).

To exemplify the workflow colon tissue was chosen as an example because the morphology is already visible in unstained tissue, whereas in lung tumours and thoracal tumours macroscopic delineation of tumours is usually not as easily achieved. In Supplementary Fig. S1B, we show an example overlay between the FTIR imaging result and the LCM image of crypts in colorectal cancer tissue samples. The samples were obtained from the Institute of Pathology, Ruhr University Bochum. The whole tissue classification is shown in Fig. S2. The figure demonstrates that all tissue types are classified simultaneously by FTIR imaging, not only the cancerous regions. The crypts (orange) upper panel and the cancerous region (red) lower panel in Fig. S1B are well recognized by our random forest classifier specific for colon tissue^34^. Finally, the transferred ROIs are precisely dissected from the sample by LCM. The whole tissue characterization and ROI selection were done without any visual inspection by a clinical expert.

### FTIR imaging and IHC of diffuse malignant mesothelioma subtyping

The differentiation of the three DMM subtypes (epithelioid, sarcomatoid, and biphasic) and the exclusion of other neoplasms are based on histopathology and an IHC marker panel. The panel includes: DPAS (special stain for mucins), CKMNF116, WT-1, CK5/6, and Calretinin. DPAS is a marker to distinguish between mesotheliomas and neoplasms with mucin production (e.g. adenocarcinoma of the lung or colon). Sarcomatoid mesotheliomas are CKMNF116 (cytokeratines 5, 6, 8, 17, and 19) and WT-1 positive. In epithelioid mesotheliomas CK5/6 (cytokeratines 5 and 6), and Calretinin are additionally seen^11^,^38^,^39^. Further markers for carcinomas as CEA, BerEP4 and TTF1 were used in the diagnostic process done before. Because these protein markers are well established, we used them as proof-of-principle (positive controls) to demonstrate the accuracy of the approach presented here.

The FTIR imaging classifier set we used here for tissue annotation of label-free, native DMM thin-sections from pleura parietalis and visceralis extended our previously published classifier for lung and thoracal tumours^36^. The classifier for DMM subtypes was trained exclusively for this study on newly acquired tissue samples. The samples must be acquired very recent due to tissue degradation which influences the proteome analysis. The classifier set is able to analyse the whole tissue heterogeneity simultaneously as mentioned above (Fig. 1). FTIR imaging replaced here several IHC markers and other histological stains at once. Usually several markers have to be used to differentiate DMM subtypes from other neoplasms of the lung or other primary sites. For the extended subtype characterization of DMM conducted here, the accuracy was 88% (specificity = 75%, sensitivity = 100%) relative to the clinical diagnoses of 17 samples from 14 independent patients (see also Table S2). The patients are all male between 36 and 83 years old with a DMM in the pleura parietalis or visceralis. Here, the sample set is actually small because mesothelioma is a rare and heterogeneous disease^13^, but our previous work has shown that the method is very robust, even with such small sample number, due to the high number of spectral markers per sample (~10 Mio) high specificity and sensitivity is obtained^34^,^35^,^36^.

In the presented study epithelioid and sarcomatoid pleural DMM tissue samples from nine patients were automatically characterized. ROIs of these two subtypes were separated from other neoplasms and collected using FTIR-guided LCM. Following the pathologists’ previous diagnosis, we collected samples from four sarcomatoid and six epithelioid DMMs. Even though the clinical diagnosis of each patient was known in advance, the annotation of each sample was performed in a blinded manner by FTIR imaging. The groups could not be equalized because of the rarity of DMM and its natural incidence of subtypes.

### Quantitative proteome analysis on diffuse malignant mesothelioma

**Figure 4:**
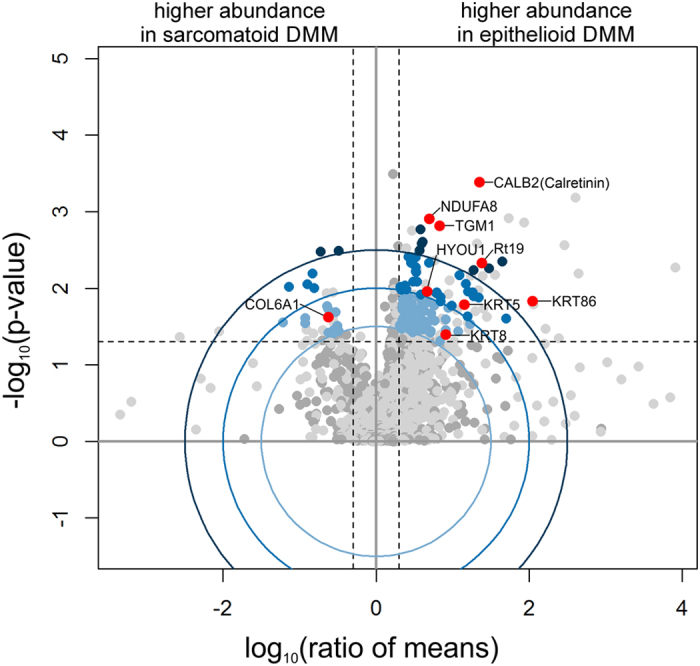
Volcano plot representing the results of the proteome analysis of epithelioid vs. sarcomatoid diffuse malignant mesothelioma. Dark grey points represent proteins quantified with minimum of two unique peptides. Light grey points represent proteins quantified with one unique peptide. Blue points represent proteins passing the Euclidian distance levels of d_eucl = 1 (light blue), 1.5 (medium blue), and 2 (dark blue); the boundaries are indicated by corresponding blue circles. Dashed lines represent the applied significance thresholds of a t-test p-value ≤0.05 (unpaired, two-sided, assuming equal variances) and an absolute fold change ≥2. Highlighted (red) proteins are part of the established protein biomarker panel or were previously described to be differentially abundant between these tissue types. Calretinin (CALB2) seems the best biomarker. Details are shown in Table 1 and Table S3.

The harvested DMM samples were provided to subsequent proteome analysis. In total, 15,432 peptides were identified, which corresponded to 2,925 protein groups (3,317 proteins). Among these, 1,869 proteins were quantified by the Progenesis QI software with at least one unique peptide (1178 with at least 2 unique peptides), and 142 proteins had significant differences in abundance between the two groups. 120 of these proteins were more abundant in epithelioid tumours, while 22 proteins were more abundant in sarcomatoid tumours. The differentially abundant proteins were ranked according to the Euclidian distance (Fig. 4, Table S3).

### Comparison with the clinical immunohistochemistry panel

**Figure 5:**
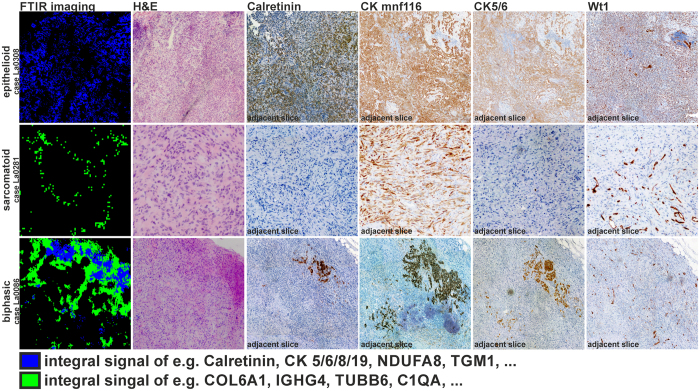
FTIR imaging and immunohistochemical staining of thin-section epithelioid, sarcomatoid and biphasic diffuse malignant mesothelioma. The correlations between FTIR imaging, histopathology (H&E), and the established IHC panel in the three subtypes of DMM are shown. FTIR imaging characterizes an integral signal of the subtle structural and molecular composition of the underlying tissue. This allows the concurrent characterization of the whole tissue heterogeneity. Here, only the subtype classes of DMM are shown in blue (epithelioid) and green (sarcomatoid). All other characterized tissue types were shielded (black) for clarity. DAB (3,3′-diaminobenzidine) staining was used to make the antibody stain of Calretinin, CKMNF116, CK5/6, and WT1 visible. The brownish colouring indicates protein expression. Counter staining was done with Haematoxylin (blueish colouring). WT-1 is used to separate mesothelioma from other cancers in lung so it is positive for all cases. For case La0308 (top row), FTIR imaging confirms the primary histopathological diagnosis of epithelioid DMM (blue). The positive reactions of CKMNF116, CK5/6, and Calretinin further confirm this diagnosis. Case La0281 (middle row) is correctly classified as sarcomatoid DMM (green) by FTIR imaging and confirmed by histopathological testing. The IHC confirms this classification with positive results for CKMNF116 and negative results for CK5/6 and Calretinin as expected. Case La0086 (bottom row) was originally classified as epithelioid DMM according to histopathological diagnostics, but the following FTIR imaging characterizes this patient as biphasic, this is confirmed by the IHC panel. The deviation is caused because the adjacent thin-section was histopathological characterized. It shows nicely the advantage of the label-free approach.

The proteome analysis revealed that the already known markers in the clinical IHC panel could all be detected by this approach. Calretinin (CALB2) was found to be the most significant marker protein to distinguish between epithelioid and sarcomatoid DMM (Fig. 4) as expected. Cytokeratine 5, 6, 8, and 19 were more abundant in epithelioid DMMs, which corresponds to the results from IHC with antibodies for CK5/6 and CKMNF116 (Table 1). In Fig. 5, the correlation between the integral FTIR imaging results, histopathology, and IHC is shown for three cases. Only the subtype characterization is shown for FTIR imaging (epithelioid in blue and sarcomatoid in green). Tissue in the black regions was characterized as different tissue types (e.g. inflammation or necrosis) and is not shown for clarity. For all three cases (La0308, La0281, and La0086), the results of the IHC panel are in nice agreement with the FTIR imaging. In case La0086, the clinical diagnosis on an adjacent section was epithelioid DMM, but in contrast FTIR imaging classified it as biphasic, with a majority of sarcomatoid cells. With the conventionally used IHC panel alone this diagnosis is difficult due to the missing IHC marker for sarcomatoid DMM.

## Discussion

Our novel approach of FTIR-guided LCM integrates label-free, automated, non-destructive tissue imaging of heterogeneous tissues and LCM to facilitate harvesting homogeneous sample material for proteome analysis. Here, all tissues were annotated by FTIR imaging, which replaces several histological stains and IHC markers at once^34^,^36^. Using a robust, objective, and automated workflow, we demonstrated that FTIR-guided LCM effectively resolved tissue heterogeneity and collected homogeneous tissue samples without any previous labelling or other treatment for subsequent proteome analysis. This approach minimizes inter- and intra-observer variability and allows easy translation to other study centres, as we demonstrated recently in collaboration with Cireca Theranostics, LLC (Cambridge, MA, USA)^40^. The robustness and objectivity of the method becomes even more evident for differential diagnoses such as tumour subtyping, grading, or assessing the heterogeneity. Conventionally, localizing diagnostically relevant subregions within tissues involves visual annotation by a human expert, which even after highly standardized training underlies subjectivity and is thus limited in terms of reproducibility. Furthermore, FTIR-guided LCM enables automated collection of samples with homogenous protein concentration using the integral of an infrared absorbance band, which is much more precise than collecting areas of the same size, allowing for more accurate quantification of relative protein abundance. This directly addresses tumour heterogeneity for protein biomarkers, which tend to be severely affected by varying cell density between tumour types and subtypes.

As a proof-of-concept, we conducted proteome analysis on samples collected using FTIR-guided LCM to identify subtypes of DMM. We were able to successfully identify all DMM biomarkers used in established diagnostic IHC panels to differentiate between epithelioid and sarcomatoid subtypes of DMM. We identified all the proteins on the IHC panel in varying abundances for all patients, despite the relatively small number of patients in our study. One previously misclassified case, La0086, demonstrated the robustness and objectivity of our method for resolving molecular changes in the tissue. Severe visual inspection of several stains by a skilled pathologist is needed to reach the same level of differential diagnosis FTIR imaging provides in one step (Fig. 5). By combination with proteome analysis the FTIR imaging approach is now also validated at the protein level and the IR patterns can now be correlated directly to biomarker panels. Thus, FTIR-guided LCM opens new avenues for ROI identification in biomarker research. This emphasizes the value of the presented approach.

Beside the established IHC panel proteins, we found other proteins previously described as overexpressed in epithelioid DMMs including HYOU1, NDUFA8, and TGM1. HYOU1 was previously described as potential serum marker for mesothelioma^41^. NDUFA8 was observed in bladder cancer previously^42^. Finally, TGM1 was described as a marker that correlates with the number of pack-years for smokers with lung adenocarcinomas^43^. In sarcomatoid DMM, COL6A1 was previously presented as a potential marker for mesothelioma^44^,^45^,^46^. We could reliably quantify all of these proteins using FTIR-guided LCM with subsequent proteome analysis using LC-MS/MS: HYOU1, NDUFA8 and TGM1 were each significantly overexpressed in epithelioid samples, and COL6A1 was significantly overexpressed in sarcomatoid samples (Table 1). Moreover, we detected many additional proteins showing significant differential expression in sarcomatoid or epithelioid DMMs, constituting valuable candidate markers to be validated on a larger number of samples.

Summing up, these findings show that FTIR-guided LCM can be translated effectively to resolve tumour and tissue heterogeneity for proteome analysis of neoplastic tissue in a very reliable, robust, and objective manner while minimizing inter- and intra-observer variability. It is straightforward to translate our approach to other entities. The FTIR spectra can now directly be compared to protein biomarker panels. In fact, we are currently investigating the use of FTIR-guided LCM in preliminary studies on colorectal cancer, bladder cancer, neurodegenerative diseases and muscle dystrophies. Furthermore, this novel approach is easily transferrable to other label-free vibrational microspectroscopy approaches such as Raman and Coherent Anti-stokes Scattering (CARS), which provide an even higher spatial resolution of few hundred nanometres^47^,^48^,^49^. In addition, FTIR-guided LCM can be combined with other *omics* techniques, most notably next generation sequencing (NGS) for studying the spatial heterogeneity of the genome and the transcriptome. Finally, the ability of FTIR-guided LCM to reproduce clinically established markers on a small number of patients is a clear indicator that resolving heterogeneity is an essential, possibly even indispensable, step for biomarker discovery. In the light of the generally weak reproducibility observed for numerous biomarker studies, it appears that being pedantic about identifying highly location specific tissue substructures is a key step for identifying reliable biomarkers. Employing label-free microscopy with high spatial resolution is in turn the key for achieving location specificity in a highly reproducible manner. Future studies involving additional clinically relevant settings are planned. The low inter- and intra-observer variability of FTIR-guided LCM will allow collection of fewer samples for biomarker studies in multicentre omics studies. Furthermore, recent developments of Quantum Cascade Laser (QCL) based IR microscopes will improve the approach further by speeding up the IR image analysis^50^. Future applications beside the sample collection for omics studies in biomarker research will be therapy progress screening in drug development. The novel FTIR-guided LCM approach combined with high-resolution proteome analysis paves the way to more specific and predictive protein biomarkers.

## Materials and Methods

### Ethical note

All methods, experiments, and experimental protocols were approved following requirements of institutional review boards (registration number 4552-12, Ethics Commission, Faculty of Medicine, Ruhr-University Bochum and registration number 13-5420-BO, Ethics Commission, University Duisburg-Essen). Furthermore, informed consent of participants was obtained following requirements of the institutional review boards and all associated methods were conducted in accordance with approved guidelines and regulations for human experimental research.

### Sample preparation

Diffuse malignant mesothelioma (DMM) samples of the pleura parietalis and visceralis were harvested within the context of surgical interventions following standard operating procedures (SOP). After harvesting, the tissue samples were cooled to 4 °C as rapidly as possible and transported within about 10 minutes to the pathologist, who determine which tissue material should be used for diagnostics and for this study. Diagnostic purposes took priority. The tissue sections were washed with isotonic saline and frozen for sectioning in a cryostat. The longest allowed period between harvest and cryogenic freezing was 30 minutes. The 10-μm thin tissue sections were deposited on polyethylene terephthalate (PET) frame slides. For every new sample, the cryostat was cleaned and the blade was changed to minimize the carryover of cells between different specimens. Afterwards, the samples were stored at −80 °C. All preparatory steps were completed as quickly as possible and are well documented. The SOP guaranteed high-quality samples with very low degradation of the tissue and its proteins, DNA, and RNA. It was developed with careful consideration of molecular biological standards.

### FTIR imaging of human tissue

Infrared hyperspectral data acquisition was completed in transmission mode using an Agilent system (Santa Clara, California, USA), consisting of a Cary 620 infrared microscope in combination with a Cary 670 FTIR spectrometer. Spectral data were collected by a mounted liquid nitrogen cooled focal plane array (FPA) MCT detector with 128 × 128 elements, providing a field of view of approximately 715 × 715 μm. The Fourier transformation was conducted using Agilent Resolution Pro Software with Mertz phase correction, a Blackman-Harris-4-term apodization, and a zero filling of 2. The spectra were saved from 2700–950 cm^−1^ with a spectral resolution of 4 cm^−1^.

The resulting raw spectral maps were pre-processed using the previously described workflow^34^. Strong disrupting signals, possibly arising from cracks or folds in the tissue, were eliminated by quality control based on the signal-to-noise ratio and the integral of the amid I band. The remaining spectra were subjected to a Mie and Resonance-Mie correction based on extended multiplicative scattering correction (EMSC) in the wavenumber range 2300–950 cm^−1^ 51,52,53,54,55. The correction was made with only one iteration step, but higher numbers of iterations (up to 20) were tested due to low scattering effects; the number of iterations did not alter the final classification. During the last step, the spectra were smoothed by a 9-point Savitzky Golay filter, providing second derivative spectra for unsupervised multivariate methods (e.g. hierarchical clustering, k-means clustering)^56^. The resulting index colour images were compared to histopathology or IHC. The resultant spectral database was used for training of a supervised classifier. In this study, a random forest (RF) classifier was used^37^. For the classification the spectra were not smoothed, and the second derivative was not calculated. The analysis was performed on the fingerprint region from 1800–950 cm^−1^.

### Histological staining

Some tissue samples were stained with Haematoxylin and Eosin after FTIR-spectroscopic measurement. The use of the same samples allows more precise overlays between the spectral image and the classical stained image. For the staining the tissue samples were washed with Milli-Q water, stained 50 seconds with Harris Haematoxylin (VWR, Germany), washed with water, counterstained with eosin (Merck, Germany), dehydrated with increasing gradients of alcohol, and mounted with Euparal (ROTH, Germany). The stained sections were imaged automatically using an Olympus BX43 microscope.

### Immunohistochemistry on DMM samples

Formaldehyde-fixed and paraffin-embedded tissue specimens were processed for immunohistochemistry using standard methods and stained using a fully automated staining instrument, BenchMark Ultra with OptiView DAB IHC Detection Kit (Ventana Medical Systems, Tucson AZ, USA) following the manufacturer’s instructions. Antibodies were used as evaluated previously due to internal quality management guidelines according to DIN EN ISO 9001 (Table S1).

### Coordinate transfer

To combine FTIR imaging results with LCM, the coordinates of the FTIR imaging microscope had to be transferred to the LCM microscope (Fig. 3). This was done by a 2-dimensional Helmert transformation, normally used in geodesy, based on three reference points. These reference points were cut into the PET foil with the LCM. After measurement, the coordinates were taken with the FTIR imaging microscope. The coordinates of the centre of the FTIR measurement were known. After transferring the slide to the LCM, the reference coordinates were obtained for this instrument, enabling the transfer of ROIs from FTIR images to shapes for LCM.

To correct for optical aberrations that can occur while switching between the two microscopes, the method was calibrated using a 1951 USAF glass slide resolution target (USAF 1951 1X, Edmund Optics, NJ, USA). This approach enabled correction of variation in pixel size and rotation between the instruments. Results of this calibration step are shown in the Supporting information (Fig. S1). For the Agilent FTIR imaging system, the picturing pixel size of the focal plane array detector was determined to be 5.65 × 5.75 μm. Furthermore, the rotation between the visible and the infrared image of the FTIR imaging microscope was ~0.06°.

### Selection of regions of interest and laser capture microdissection

The results of the random forest (RF) classification were represented in an index colour image. Each colour represented a different tissue type, and only the colour of the tissue type of interest was considered as region of interest (ROI). The middle of each marginal pixel of these ROIs was used as a coordinate for the LCM shapes. Due to technical issues, such as lifting of the sample (Zeiss Palm) or field of view (Leica LMD7), the shapes were size limited. A watershed algorithm was used to split larger areas into smaller regions. These smaller regions could then be cut by LCM. Collected sample size was classically defined by area (μm^2^). The FTIR-guided LCM method allowed use of the integral of a spectral band (e.g., amid 1) for the collection of sample pools with nearly equal protein concentration. The samples were collected in 15 μl of 50 mM ammonium bicarbonate with 0.1% RapiGest SF surfactant (Waters GmbH, Eschborn, Germany). For microdissection a Leica LMD7 and a Zeiss Palm system were used. The samples were stored at −80 °C for further processing.

### Tryptic digestion

The laser capture microdissected samples were sonicated in an ultrasonic bath for 1 min on ice. Subsequently, the samples were incubated for 5 min at 95 °C and the protein disulphide bonds were reduced with 5 mM DTT for 30 min at 60 °C. Alkylation was performed for 30 min at room temperature in the dark using 15 mM iodoacetamide. The proteins were digested overnight at 37 °C using 20 ng trypsin (SERVA Electrophoresis, Heidelberg, Germany) per sample. The enzymatic digestion was stopped by acidification with 0.5% TFA for 30 min at 37 °C, and precipitated RapiGest was removed by centrifugation. The samples were vacuum-dried and subsequently dissolved in 0.1% TFA. Each 1-mm^2^ sample of microdissected tissue was divided in three technical replicates.

### LC-MS/MS analysis

The RPLC−MS/MS analysis was conducted on an Ultimate 3000 RSLCnano system (Thermo Scientific, Bremen, Germany) coupled online to an Orbitrap Elite mass spectrometer (Thermo Scientific, Bremen, Germany). The peptides were injected in a volume of 15 μl and pre-concentrated with 0.1% TFA on a trap column for 7 min (Acclaim PepMap 100, 300 μm × 5 mm, C18, 5 μm, 100 Å; flow rate 30 μl/min). Subsequently, the peptides were separated on the analytical column (Acclaim PepMap RSLC, 75 μm × 50 cm, nano Viper, C18, 2 μm, 100 Å) by a gradient from 5% to 40% solvent B over 98 min (solvent A: 0.1% FA, solvent B: 0.1% FA, 84% acetonitrile; flow rate 400 nl/min; column oven temperature 60 °C). The instrument was operated in a data-dependent mode. Full scan mass spectra were acquired in profile mode at a resolution of 60,000 at 400 m/z in the Orbitrap analyzer and within a mass range of 350–2000 m/z. The 20 most abundant precursors were selected for MS/MS analysis. The tandem mass spectra were measured in the linear ion trap after peptide fragmentation by collision-induced dissociation. The precursor isolation width was 1.0 m/z. Charge state screening was enabled and only precursors of charge states +2, +3 and +4 were fragmented with normalized collision energy of 35.0%. The mass range for MS/MS spectra was 0–2000 m/z and the scan mode was centroid. Dynamic exclusion parameters were used: Exclusion list size was 500, exclusion duration was 30 s, and the mass width relative to excluded mass was ± 10 ppm. The time for one duty cycle was 3.6 s if 20 MS/MS spectra per full scan were acquired.

### Peptide identification

Peptide identification was conducted using Proteome Discoverer Software (ver. 1.4.1.14, Thermo Fisher Scientific, Rockford, IL, USA). The mass spectra were searched against UniProtKB/Swiss-Prot database (Uniprot/Swissprot-Release 2015_05 of 29.04.2015; 548,454) restricted to *Homo sapiens* using the Mascot search engine (version 2.5). The mass tolerance was set to 5 ppm for precursor ions and 0.4 daltons for fragment ions. The algorithm considered up to one tryptic missed cleavage, as well as chemical modifications of methionine (oxidation, dynamic) and cysteine (carbamidomethyl, static). The percolator function implemented in Proteome Discoverer was used to estimate peptide confidence, and only peptides that passed with a false discovery rate < 1% (q-value < 0.01) were considered for analysis.

### Protein quantification

Ion intensity-based label-free quantification was performed using Progenesis QI for proteomics (ver. 2.0.5387.52102, Nonlinear Dynamics Ltd., Newcastle upon Tyne, UK). To account for retention time shifts, the LC-MS runs were aligned to one run chosen automatically by the software. A master list of features considering retention time and m/z was generated considering peptide ions with minimum three isotopic peaks and charges states +2, +3 and +4. The peptide identifications from Proteome Discoverer were imported into the software and matched to the respective features. The abundance for each protein was calculated considering the normalized ion intensities of all non-conflicting peptides of a protein. Differences in protein abundance between the experimental groups were assessed by means of Student’s t-test (two-sided, assuming equal variances) using arcsinh-transformed data. The arcsinh-transformed values were averaged for the three technical replicates, and these mean values were used for subsequent calculations. The fold changes were determined on the scale of normalized protein abundance. Generally, proteins quantified using a minimum of two unique peptides, a fold change ≥2 or ≤−2, and a *p*-value ≤0.05 were considered to be significantly differentially expressed. To ensure a meaningful ranking of the protein lists, we calculated the Euclidian distance:


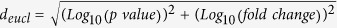


This accounts for both the p-value and the fold change. The mass spectrometry proteomics data presented in this manuscript have been deposited to the ProteomeXchange Consortium via the PRIDE partner repository and are available with the dataset identifier PXD004818 and 10.6019/PXD004818.

## Additional Information

**How to cite this article:** Großerueschkamp, F. *et al*. Spatial and molecular resolution of diffuse malignant mesothelioma heterogeneity by integrating label-free FTIR imaging, laser capture microdissection and proteomics. *Sci. Rep.* **7**, 44829; doi: 10.1038/srep44829 (2017).

**Publisher's note:** Springer Nature remains neutral with regard to jurisdictional claims in published maps and institutional affiliations.

## Supplementary Material

Supplementary Information

## Figures and Tables

**Table 1:** Examples of proteins that were significantly more abundant in epithelioid or sarcomatoid DMM.

UniProt Accession	Gene	Protein	p value	Fold Change	d_eucl_
P22676	CALB2	Calretinin	0.0004	22.4	3.64
O43790	KRT86	Keratin, type II cuticular Hb6	0.0148	110.5	2.74
P08727	RT19	Keratin, type I cytoskeletal 19	0.0047	24.2	2.71
P13647	KRT5	Keratin, type II cytoskeletal 5	0.0232	15.7	2.03
P05787	KRT8	Keratin, type II cytoskeletal 8	0.0484	6.9	1.56
P51970	NDUFA8	NADH dehydrogenase [ubiquinone] 1 alpha subcomplex subunit 8	0.0012	4.9	2.99
P22735	TGM1	Protein-glutamine gamma-glutamyltransferase	0.0015	6.7	2.93
Q9Y4L1	HYOU1	Hypoxia up-regulated protein 1	0.0120	3.8	2.01
P12109^*^	COL6A1	Collagen alpha-1(VI) chain	0.0238	4.2	1.74

^*^Grey shading indicates that this protein was more abundant in sarcomatoid tissue
