# Supplementary material for: Spatial and molecular resolution of diffuse malignant mesothelioma heterogeneity by integrating label-free FTIR imaging, laser capture microdissection and proteomics
Source: Sci Rep. 2017 Mar 30;7:44829. doi: 10.1038/srep44829 (PMC5372163; doi:10.1038/srep44829)
Supplement: Supplementary Information [file srep44829-s1.pdf]

Spatial and molecular resolution of diffuse malignant mesothelioma heterogeneity by integrating label-free FTIR imaging, laser capture microdissection and proteomics

Authors:

Frederik Großerueschkamp[1], Thilo Bracht[2], Hanna C. Diehl[2], Claus Kuepper[1], Maike Ahrens[2], Angela Kallenbach-Thieltges[1], Axel Mosig[1], Martin Eisenacher[2], Katrin Marcus[2], Thomas Behrens[3], Thomas Brüning[3], Dirk Theegarten[4], Barbara Sitek[2#], Klaus Gerwert[1#*]

Affiliations:

[1] Ruhr-University Bochum, Department of Biophysics, Bochum, Germany

[2] Ruhr-University Bochum, Medizinisches Proteom- Center (MPC), Bochum, Germany

[3] Institute for Prevention and Occupational Medicine of the German Social Accident Insurance, Institute of the Ruhr Universität Bochum (IPA), Bochum, Germany

[4] University Duisburg Essen, University Hospital Essen, Institute of Pathology, Essen, Germany

*To whom correspondence should be addressed: Klaus Gerwert: gerwert@bph.rub.de

[#]Theses authors contributed equally to the work

List of Supplementary Materials

Fig. S1: Calibration and accuracy of coordinate transfer methods.

Fig. S2: FTIR imaging results for the whole, label-free sample colon tissue sections.

Table S1: Antibodies used for immunohistochemistry.

Table S2: Validation for using the random forest classifier for 17 samples from 14 patients.

Table S3: Proteins with significant differential abundance between epithelioid and sarcomatoid DDM subtypes.

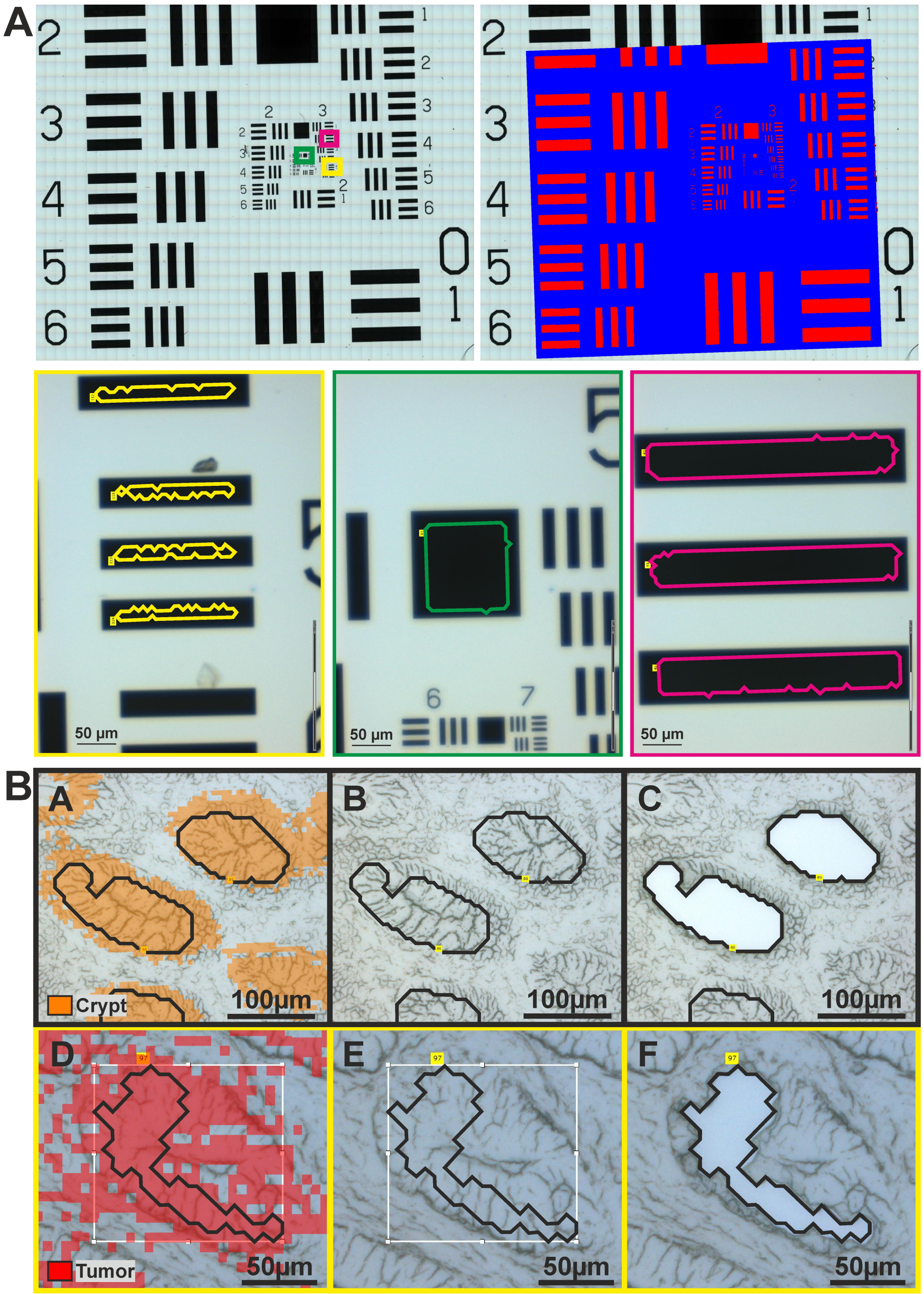

Figure S1. Calibration and accuracy of coordinate transfer methods. A) The high accuracy (~10 µm) of the coordinate transfer is shown with an USAF (United States Air Force) target. The target is also used for calibrating the coordinate transfer between the two modalities: FTIR imaging and LCM. This includes rotation and distortion between the modalities used which can

be seen by the overlay of the FTIR index colour image with the USAF target on the upper right side (blue and red). The red regions were chosen as ROI. The resulting cutting shapes for LCM were shown in detail at the bottom of the figure. **B)** Accuracy of cutting shapes from FTIR imaging is shown with examples of crypts and precancerous crypts in colon. Colon was chosen to demonstrate the process because the structure is readily visible in thin-sections of native tissue. These samples were obtained from the Institute of Pathology, Ruhr University Bochum. A. Overlay of the spectral classification of healthy crypts with the transferred cutting shape on the LCM. B. Cutting shape for healthy crypts. C. ROIs cut out with high precision. D. Overlay of the spectral classification of a cancerous lesion with the transferred cutting shape on the LCM. E. Cutting shape for colorectal cancer. F. ROIs cut out with high precision, even in this more complicated case.

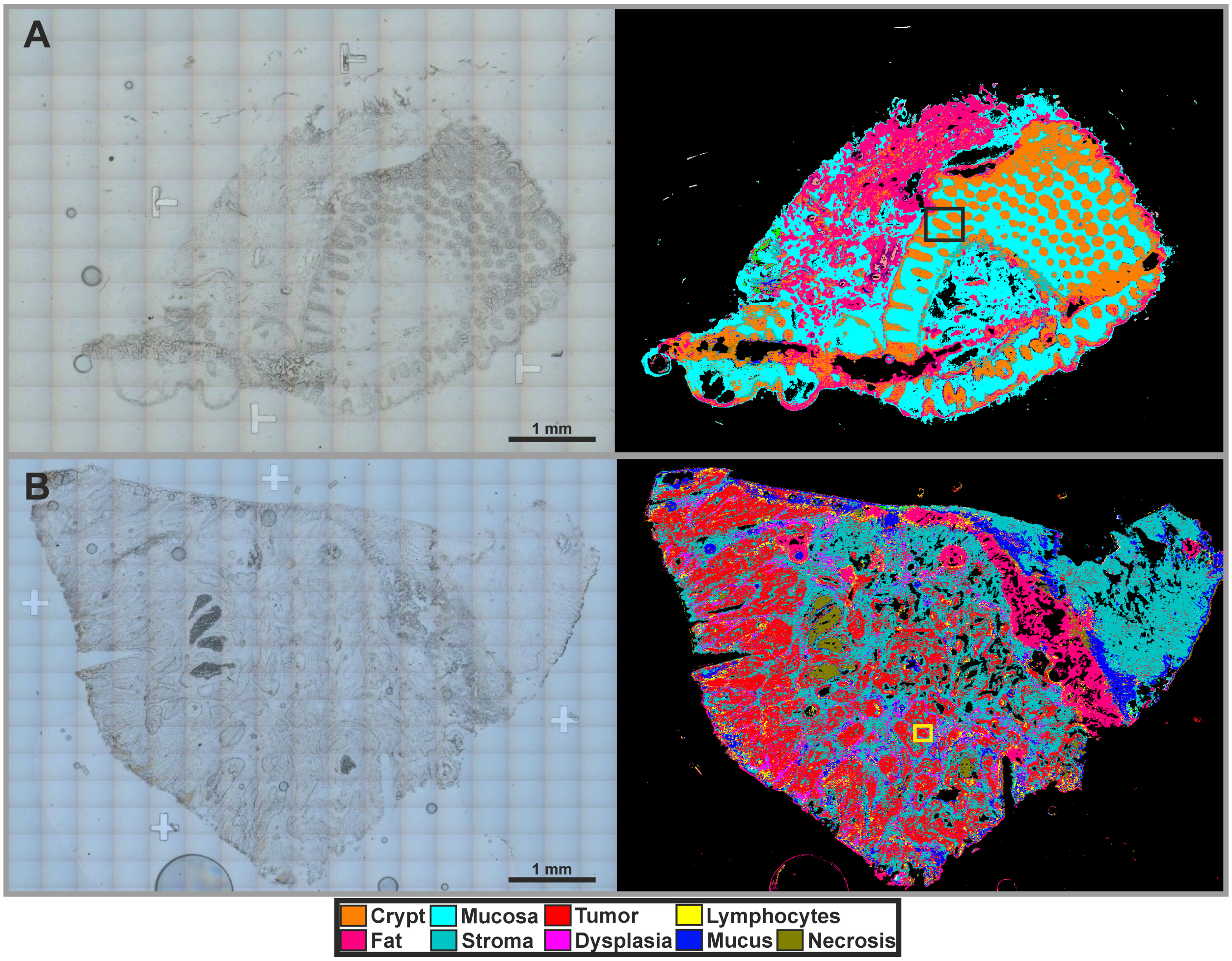

Figure S2. FTIR imaging results for the whole, label-free sample colon tissue sections. The black and yellow squares correspond to the detailed images presented in **Fig. 3**. The image illustrates that all tissue types can be differentiated automatically by FTIR imaging.

Table S1: Antibodies used for immunohistochemistry

Antibody	Company	Clone	Dilution
Calretinin	DAKO	DAK-Calret1	1:600
CKMNF116	DAKO	CKMNF116	1:1000
CK5-6	DAKO	D5/16B4	1:500
WT1	DAKO	6F-H2	1:400

Table S2: Validation for using the random forest classifier for 17 samples from 14 patients

	epithelioid	sarcomatoid
**true**	9	6
**false**	2	0

Table S3: Proteins with significant differential abundance between epithelioid and sarcomatoid DDM subtypes

Higher abundance in epithelioid tumours

UniProt Accession	Gene	Protein	p value[1]	Fold Change[2]	d_{eucl}[3]
P22676	CALB2	Calretinin	0.0004	22.4	3.64
P51970	NDUFA8	NADH dehydrogenase [ubiquinone] 1 alpha subcomplex subunit 8	0.0012	4.9	2.99
P22735	TGM1	Protein-glutamine gamma-glutamyltransferase	0.0015	6.7	2.93
Q15847	ADIRF	Adipogenesis regulatory factor	0.0045	44.5	2.87
P49748	ACADVL	Very long-chain specific acyl-CoA dehydrogenase, mitochondrial	0.0017	3.8	2.83
O43790	KRT86	Keratin, type II cuticular Hb6	0.0148	110.5	2.74
P08727	RT19	Keratin, type I cytoskeletal 19	0.0047	24.2	2.71
P12532	CKMT1A	Creatine kinase U-type, mitochondrial	0.0055	29.7	2.70
Q8NFW8	CMAS	N-acylneuraminate cytidylyltransferase	0.0025	4.0	2.68
O60884	DNAJA2	DnaJ homolog subfamily A member 2	0.0026	3.9	2.65
O14745	SLC9A3R1	Na(+)/H(+) exchange regulatory cofactor NHE-RF1	0.0058	18.7	2.58
O00159	MYO1C	Unconventional myosin-Ic	0.0032	3.7	2.56
P35221	CTNNA1	Catenin alpha-1	0.0039	3.3	2.46
P17931	LGALS3	Galectin-3	0.0039	2.7	2.45
P52943	CRIP2	Cysteine-rich protein 2	0.0046	4.9	2.43
Q13228	SELENBP1	Selenium-binding protein 1	0.0067	12.2	2.43
P05023	ATP1A1	Sodium/potassium-transporting ATPase subunit alpha-1	0.0047	2.9	2.38
O94788	ALDH1A2	Retinal dehydrogenase 2	0.0087	14.8	2.37
P47712	PLA2G4A	Cytosolic phospholipase A2	0.0249	50.0	2.34
O60716	CTNND1	Catenin delta-1	0.0053	3.3	2.33
Q9H773	DCTPP1	dCTP pyrophosphatase 1	0.0113	18.2	2.32
Q02952	AKAP12	A-kinase anchor protein 12	0.0132	21.8	2.31
O60437	PPL PE	Periplakin	0.0110	15.8	2.29
O95833	CLIC3	Chloride intracellular channel protein 3	0.0126	18.7	2.29

Q9NUP9	LIN7C	Protein lin-7 homolog C	0.0060	3.3	2.28
P53985	SLC16A1	Monocarboxylate transporter 1	0.0061	3.3	2.27
P36871	PGM1	Phosphoglucomutase-1	0.0082	3.4	2.15
P63151	PPP2R2A	Serine/threonine-protein phosphatase 2A 55 kDa regulatory subunit B alpha isoform	0.0082	3.0	2.14
Q8WUT4	LRRN4	Leucine-rich repeat neuronal protein 4	0.0165	14.4	2.12
O76027	ANXA9	Annexin A9	0.0112	6.1	2.10
Q13263	TRIM28	Transcription intermediary factor 1-beta	0.0117	6.1	2.09
P11766	ADH5	Alcohol dehydrogenase class-3	0.0092	2.4	2.07
Q92896	GLG1	Golgi apparatus protein 1	0.0110	4.6	2.07
Q05707	COL14A1	Collagen alpha-1(XIV) chain	0.0131	6.9	2.06
P50995	ANXA11	Annexin A11	0.0096	2.1	2.04
P78347	GTF2I	General transcription factor II-I	0.0170	9.7	2.03
P13647	KRT5	Keratin, type II cytoskeletal 5	0.0232	15.7	2.03
Q99805	TM9SF2	Transmembrane 9 superfamily member 2	0.0104	2.5	2.02
P18077	RPL35A	60S ribosomal protein L35a	0.0102	2.3	2.02
Q16647	PTGIS	Prostacyclin synthase	0.0148	7.0	2.02
O43707	ACTN4	Alpha-actinin-4	0.0104	2.1	2.01
Q9Y4L1	HYOU1	Hypoxia up-regulated protein 1	0.0120	3.8	2.01
Q53SF7	COBLL1	Cordon-bleu protein-like 1	0.0179	8.8	1.99
P35222	CTNNB1	Catenin beta-1	0.0129	3.6	1.97
Q13087	PDIA2	Protein disulfide-isomerase A2	0.0295	16.8	1.96
P08107	HSPA1A	Heat shock 70 kDa protein 1A/1B	0.0122	2.4	1.95
P40763	STAT3	Signal transducer and activator of transcription 3	0.0151	4.7	1.94
Q9Y265	RUVBL1	RuvB-like 1	0.0129	2.7	1.94
Q9UQB8	BAIAP2	Brain-specific angiogenesis inhibitor 1-associated protein 2	0.0166	5.7	1.93
Q01469	FABP5	Fatty acid-binding protein, epidermal	0.0156	4.0	1.90
P14314	PRKCSH	Glucosidase 2 subunit beta	0.0139	2.2	1.89
P19404	NDUFV2	NADH dehydrogenase [ubiquinone] flavoprotein 2, mitochondrial	0.0149	2.5	1.87
P61956	SUMO2	Small ubiquitin-related modifier 2	0.0160	2.9	1.85
Q9UK22	FBXO2	F-box only protein 2	0.0254	8.1	1.84
Q9Y3I0	C22orf28	tRNA-splicing ligase RtcB homolog	0.0167	2.7	1.83

P55769	NHP2L1	NHP2-like protein 1	0.0213	5.2	1.82
P49959	MRE11A	Double-strand break repair protein MRE11A	0.0196	4.3	1.82
P15586	GNS	N-acetylglucosamine-6-sulfatase	0.0189	3.6	1.81
P10155	TROVE2	60 kDa SS-A/Ro ribonucleoprotein	0.0177	2.8	1.81
P54727	RAD23B	UV excision repair protein RAD23 homolog B	0.0168	2.2	1.81
Q14126	DSG2	Desmoglein-2	0.0365	11.8	1.79
Q14247	CTTN	Src substrate cortactin	0.0208	3.9	1.78
Q08257	CRYZ	Quinone oxidoreductase	0.0205	3.3	1.77
P63208	SKP1	S-phase kinase-associated protein 1	0.0189	2.2	1.76
P15311	EZR	Ezrin	0.0266	5.8	1.75
O43684	BUB3	Mitotic checkpoint protein BUB3	0.0214	3.2	1.74
Q9Y277	VDAC3	Voltage-dependent anion-selective channel protein 3	0.0207	2.7	1.74
P60903	S100A10	Protein S100-A10	0.0351	8.1	1.71
P15924	DSP	Desmoplakin	0.0276	4.8	1.70
Q8NCW5	APOA1BP	NAD(P)H-hydrate epimerase	0.0242	3.2	1.70
P51858	HDGF	Hepatoma-derived growth factor	0.0261	4.0	1.69
P28331	NDUFS1	NADH-ubiquinone oxidoreductase 75 kDa subunit, mitochondrial	0.0232	2.7	1.69
O00468	AGRN	Agrin	0.0269	4.2	1.69
P63167	DYNLL1	Dynein light chain 1, cytoplasmic	0.0247	3.0	1.68
P29972	AQP1	Aquaporin-1	0.0405	8.2	1.67
P62244	RPS15A	40S ribosomal protein S15a	0.0243	2.5	1.66
Q9BXS5	AP1M1	AP-1 complex subunit mu-1	0.0249	2.4	1.65
Q9BVK6	TMED9	Transmembrane emp24 domain-containing protein 9	0.0245	2.3	1.65
Q13813	SPTAN1	Spectrin alpha chain, non-erythrocytic 1	0.0267	2.9	1.64
Q99873	PRMT1	Protein arginine N-methyltransferase 1	0.0260	2.5	1.63
P13073	COX4I1	Cytochrome c oxidase subunit 4 isoform 1, mitochondrial	0.0267	2.8	1.63
P42704	LRPPRC	Leucine-rich PPR motif-containing protein, mitochondrial	0.02889	3.4	1.63
P13489	RNH1	Ribonuclease inhibitor	0.0260	2.2	1.62
P00966	ASS1	Argininosuccinate synthase	0.0269	2.4	1.62
P30044	PRDX5	Peroxiredoxin-5, mitochondrial	0.0276	2.6	1.61
O75131	CPNE3	Copine-3 OS=Homo sapiens	0.0362	5.2	1.61
P27694	RPA1	Replication protein A 70 kDa DNA-binding subunit	0.0342	4.2	1.59

Q9H8H3	METTL7A	Methyltransferase-like protein 7A	0.0342	4.1	1.59
P22105	TNXB	Tenascin-X	0.0380	5.1	1.59
Q08211	DHX9	ATP-dependent RNA helicase A	0.0297	2.7	1.59
Q01082	SPTBN1	Spectrin beta chain, non-erythrocytic 1	0.0318	3.3	1.58
P05787	KRT8	Keratin, type II cytoskeletal 8	0.0484	6.9	1.56
P35241	RDX	Radixin	0.0313	2.6	1.56
P51991	HNRNPA3	Heterogeneous nuclear ribonucleoprotein A3	0.0322	2.7	1.55
P62826	RAN	GTP-binding nuclear protein Ran	0.0301	2.1	1.55
P07602	PSAP	Proactivator polypeptide	0.0356	3.5	1.55
O95831	AIFM1	Apoptosis-inducing factor 1, mitochondrial	0.0315	2.0	1.53
P09874	PARP1	Poly [ADP-ribose] polymerase 1	0.0342	2.7	1.53
Q92544	TM9SF4	Transmembrane 9 superfamily member 4	0.0322	2.1	1.53
Q13151	HNRNPA0	Heterogeneous nuclear ribonucleoprotein A0	0.0333	2.2	1.52
Q9Y230	RUVBL2	RuvB-like 2	0.0336	2.2	1.51
Q8N1N4	KRT78	Keratin, type II cytoskeletal 78	0.0376	3.0	1.50
Q9Y333	LSM2	U6 snRNA-associated Sm-like protein LSm2	0.0405	3.3	1.49
Q15185	PTGES3	Prostaglandin E synthase 3	0.0363	2.2	1.48
P55287	CDH11	Cadherin-11	0.0401	3.0	1.48
P61019	RAB2A	Ras-related protein Rab-2A	0.0377	2.3	1.47
P09211	GSTP1	Glutathione S-transferase P	0.0380	2.2	1.46
Q9H8Y8	GORASP2	Golgi reassembly-stacking protein 2	0.0396	2.5	1.46
P23634	ATP2B4	Plasma membrane calcium-transporting ATPase 4	0.0457	3.7	1.46
Q96IZ0	PAWR	PRKC apoptosis WT1 regulator protein	0.0440	3.3	1.45
P42285	SKIV2L2	Superkiller viralicidic activity 2-like 2	0.0449	3.4	1.45
Q13011	ECH1	Delta(3,5)-Delta(2,4)-dienoyl-CoA isomerase, mitochondrial	0.0403	2.2	1.44
Q9UQ35	SRRM2	Serine/arginine repetitive matrix protein 2	0.0497	3.9	1.43
Q9UHX1	PUF60	Poly(U)-binding-splicing factor PUF60	0.0475	3.4	1.43
Q9BSJ8	ESYT1	Extended synaptotagmin-1	0.0415	2.2	1.42
Q9UIJ7	AK3	GTP:AMP phosphotransferase, mitochondrial	0.0433	2.6	1.42
P29590	PML	Protein PML	0.0459	2.4	1.39
P21291	CSRP1	Cysteine and glycine-rich protein 1	0.0451	2.0	1.38
Q15233	NONO	Non-POU domain-containing octamer-binding protein	0.0463	2.2	1.38
Q15843	NEDD8	NEDD8	0.0483	2.2	1.36

**Higher abundance in sarcomatoid tumors**					
**UniProt Accession**	**Gene**	**Protein**	***p* value[1]**	**Fold Change[2]**	**d_{eucl}[3]**
P01861	IGHG4	Ig gamma-4 chain C region	0.0033	5.3	2.59
Q9BUF5	TUBB6	Tubulin beta-6	0.0033	3.1	2.54
P02745	C1QA	Complement C1q subcomponent subunit	0.0064	6.8	2.34
P14207	FOLR2	Folate receptor beta	0.0096	13.8	2.32
P20908	COL5A1	Collagen alpha-1(V) chain	0.0088	7.9	2.24
P01859	IGHG2	Ig gamma-2 chain C region	0.0099	6.5	2.16
P55290	CDH13	Cadherin-13	0.0278	16.6	1.98
Q9BXX0	EMILIN2	EMILIN-2	0.0170	4.4	1.88
P35555	FBN1	Fibrillin-1	0.0245	8.5	1.86
P12111	COL6A	Collagen alpha-3(VI) chain	0.0184	4.3	1.85
P00488	F13A1	Coagulation factor XIII A chain	0.0284	8.5	1.80
P80748	NA	Ig lambda chain V-III region LOI	0.0205	3.4	1.77
P12109	COL6A1	Collagen alpha-1(VI) chain	0.0238	4.2	1.74
O43570	CA12	Carbonic anhydrase 12	0.0309	3.2	1.59
P02747	C1QC	Complement C1q subcomponent subunit C	0.0354	3.7	1.56
P21980	TGM2	Protein-glutamine gamma-glutamyltransferase 2	0.0380	4.2	1.55
P01877	IGHA2	Ig alpha-2 chain C region	0.0381	3.8	1.53
P01834	IGKC	Ig kappa chain C region	0.0360	3.1	1.53
P02746	C1QB	Complement C1q subcomponent subunit B	0.0386	3.6	1.52
P02452	COL1A1	Collagen alpha-1(I) chain	0.0476	5.1	1.50
P01857	IGHG1	Ig gamma-1 chain C region	0.0431	3.2	1.45
P01903	HLA-DRA	HLA class II histocompatibility antigen, DR alpha chain	0.0407	2.4	1.44

[1]The significance of differential abundance was tested using Student’s t-test (two-sided, equal variances)

[2]All proteins were quantified with at least two unique peptides

[3]The protein list was ranked using the Euclidian distance
